# A stage-based framework to interpret regulatory T cell biology after heart transplantation

**DOI:** 10.3389/fcvm.2026.1777360

**Published:** 2026-03-31

**Authors:** Yuansheng Wang, Mingyang Ni, Aijia Zheng, Yuqing Peng, Jinbiao Liu, Qing Xiong, Xueli Wang, Vuk Savkovic, Sini Kang

**Affiliations:** 1 Cardiodynamics and Assistive Tech Group, National “111” Center for Cellular Regulation and Molecular Pharmaceutics, Wuhan Joint Laboratory (China-Serbia) of Biomedical and Health Science, School of Life and Health Sciences, Hubei University of Technology, Wuhan, China; 2 Hubei Provincial Enterprise Technology Center, Wuhan Vickor Medical Technology Co., Ltd., Wuhan, China; 3 Merseburg University of Applied Sciences, Merseburg, Germany

**Keywords:** acute cellular rejection, cardiac allograft vasculopathy, foxp, heart transplantation, regulatory T cells

## Abstract

Regulatory T cells play a pivotal role in immune responses following heart transplantation, influencing the entire post-transplant process. This article examines Treg dynamics in a stage-specific framework and their clinical implications. In the early phase (0–30 days), dominated by injury-related sterile inflammation, Treg recruitment affects local inflammation resolution and tissue repair, potentially altering risks of early immune injury and rejection. The intermediate phase (1–6 months) features high acute cellular rejection risk with ongoing immunosuppression adjustments; Treg quantity, phenotype, and suppressive function are closely associated with the regulation of anti-donor immune responses. In the late phase (>6 months), chronic low-grade inflammation and progressive vascular remodeling predominate, where Tregs suppress persistent immune attacks but may promote fibrosis via repair pathways, exhibiting bidirectional effects. This article highlights Treg detection limitations, including *FOXP3* specificity, epigenetic stability, and blood-graft discrepancies. Future directions encompass multimarker monitoring, dynamic risk models, Treg cell therapy, and interventions like cytokine/microbiome modulation to achieve precise immunoregulation, reduce rejection, minimize complications, and improve long-term graft survival.

## 1 Introduction

Heart transplantation remains a key therapeutic option for long-term survival benefit in patients with end-stage heart failure, but post-transplant immunological challenges still significantly limit long-term graft outcomes (1). Modern immunosuppressive regimens have reduced the incidence of overt acute rejection; however, the lack of precise immune monitoring often necessitates broad-spectrum immunosuppression, which inevitably increases risks of opportunistic infections, malignancies, and organ damage. Consequently, establishing stage-specific biomarkers, such as regulatory T cells, is crucial to optimize the risk-benefit trade-off in long-term management (2, 3). Clinical management thus faces ongoing risk-benefit trade-offs: inadequate immunosuppression leads to rejection recurrence, while excessive immunosuppression causes diminished immune defense and cumulative long-term toxicity. Meanwhile, chronic immune-mediated injury and allograft vasculopathy remain major causes of late graft failure, indicating that reliance on broad-spectrum immunosuppression alone cannot meet long-term management needs (4). Achieving selective control over donor antigen responses while maximally preserving overall immune defense capacity remains a critical direction for advancement in transplantation.

Regulatory T cells (Tregs) are a critical CD4+ T cell subset responsible for maintaining immune homeostasis. In general, Tregs limit excessive immune responses to donor antigens through mechanisms including suppression of effector T cell responses, modulation of antigen-presenting cells, and secretion of immunosuppressive cytokines, thereby providing a theoretical foundation for graft protection and long-term tolerance (5). However, metrics of Treg detection do not necessarily equate to their stability or suppressive function (6). In peripheral blood, Treg counts or *FOXP3* expression levels are highly susceptible to influences from activation status, cellular distribution, and assay platforms, making single time-point measurements often insufficient to directly correlate with clinical outcomes (7).

The immune response following heart transplantation exhibits a distinct temporal structure. The early post-transplant period is dominated by sterile inflammation driven by surgical trauma and ischemia-reperfusion injury (8); followed by a phase of relatively high acute rejection risk and gradual adjustment of immunosuppression (9); whereas the late period is characterized predominantly by the cumulative effects of chronic low-grade inflammation, persistent endothelial activation, and vascular remodeling (10). Treg numbers, phenotype, and function vary according to these stage-specific microenvironments and are collectively shaped by multiple factors, including immunosuppressive regimens, infection/inflammatory events, and tissue homing conditions (11). Mixing data from different time periods for interpretation can readily lead to apparently contradictory conclusions, thereby undermining the reproducibility and translational potential of Treg-related monitoring and intervention strategies.

To address the aforementioned issues, this review organizes its content according to the post-transplantation timeline, examining Treg quantitative changes and tissue distribution characteristics across three distinct phases after heart transplantation: 0–30 days, 1–6 months, and beyond 6 months; with particular emphasis on the impact of inflammatory signals, costimulatory pathways, and metabolic stress on Treg stability and suppressive function, as well as their associations with acute cellular rejection (ACR) and processes related to late allograft vasculopathy. Subsequently, this review delineates the boundaries of applicability for Treg-related detection markers, underscores potential systematic discrepancies between peripheral blood and graft tissue findings, and highlights the limitations of *FOXP3*-based measurements regarding specificity and functional inference. Beyond summarizing biological mechanisms, this review aims to provide clinicians with an integrated, time-stratified framework. This framework is designed to aid in interpreting discordant Treg readouts and to guide the selection of appropriate monitoring tools for risk assessment at different post-transplant stages.

## 2 Search strategy

A comprehensive literature search was conducted using PubMed and Web of Science databases up to October 2025. Key search terms included ‘Regulatory T cells’, ‘Heart Transplantation’, ‘Acute Cellular Rejection’, ‘Cardiac Allograft Vasculopathy’, and ‘Ischemia-Reperfusion Injury’. We prioritized studies involving human subjects but included pivotal murine models where mechanistic insights were essential. Studies were categorized into three phases (0–30 days, 1–6 months, >6 months) based on the timing of sample collection reported.

## 3 Definition and detection of Tregs in heart transplantation

Classically, Treg are defined as CD4+ CD25+ T cells that express the transcription factor *FOXP3* (12). *FOXP3* serves as the pivotal transcription factor for Treg differentiation and function, with its essential role confirmed by human IPEX syndrome (13), which arises from *FOXP3* gene mutations (13). In addition to CD25 and intracellular *FOXP3*, low expression of the IL-7 receptor *α*-chain (CD127) has been established as an important surface phenotype for human peripheral Tregs, with reduced CD127 levels negatively correlating with *FOXP3* expression and Treg suppressive function. Consequently, the population commonly defined as CD4+ CD25high CD127low/- is used to isolate a relatively pure Treg subset, which typically constitutes approximately 5%–10% of total CD4+ T cells (14). Tregs exert immunosuppressive functions through multiple mechanisms, including secretion of inhibitory cytokines to suppress effector T cell proliferation (15); expression of the ectoenzyme CD39 to generate adenosine and other small-molecule mediators, resulting in bystander suppression of effector cells (16); release of granzymes via cytotoxic pathways and expression of death receptor ligands to directly kill effector T/B cells (17); high expression of CTLA-4 to competitively bind B7 molecules on dendritic cells and downregulate their expression, thereby attenuating costimulatory signals and inducing tolerogenic dendritic cells (18); and sustained high expression of CD25 to competitively consume IL-2, leading to apoptosis of effector T cells due to growth factor deprivation (19). These multifaceted suppressive mechanisms position Tregs as an attractive therapeutic target for preventing both acute and chronic graft rejection.

### 3.1 Markers for Treg identification and limitations of measurement

Accurate identification of human peripheral Tregs requires the integration of multiple markers, as no single marker suffices to distinguish Tregs from conventional T cells. Furthermore, a panel of auxiliary markers can be utilized for Treg identification and functional evaluation, including costimulatory molecules, coinhibitory molecules, and chemokine receptors (20). It is noteworthy that these markers are not exclusive to Tregs; nonetheless, their expression levels or combinatorial patterns in Tregs differ from those observed in most effector T cells. For instance, human Tregs highly express the key adenosine pathway enzyme CD39, with expression levels correlating with *FOXP3* stability in Tregs (21). Similarly, the transcription factor Helios was initially considered highly expressed in thymus-derived Tregs and served as an auxiliary marker for natural Tregs; however, subsequent studies have shown that Helios is not essential for maintaining stability and functions merely as a correlative marker (22). Overall, accurate delineation of the Treg population in both research and clinical settings necessitates the combined use of multiple surface and intracellular markers.

### 3.2 Role and limitations of *FOXP3*

*FOXP3* serves as both a hallmark marker and a key maintainer of Treg function; however, its use in evaluating human Tregs is subject to certain limitations (23). First, specificity issues arise because activated conventional human CD4+ T cells can transiently express *FOXP3* under strong stimulation, yet these cells lack suppressive function (24). Consequently, *FOXP3* expression alone cannot fully distinguish bona fide Tregs from activated effector T cells that transiently express *FOXP3*. Similar phenomena have been observed in the transplantation setting: during acute rejection, FOXP3 gene expression in the graft is markedly elevated. While this suggests the presence of FOXP3-expressing cells, it requires careful interpretation as it may reflect either Treg infiltration or activation-induced upregulation in effector T cells. However, these *FOXP3*+ cells are not necessarily functional Tregs; some may represent activated T cells expressing *FOXP3* due to intense immune activation (25). Studies have shown that in heart transplant patients experiencing acute rejection, peripheral CD4+ CD25bright *FOXP3*+ T cells exhibit higher CD127 expression levels, suggesting that these purported Tregs may be in an activated or unstable state with impaired suppressive capacity (26). This indicates that *FOXP3*+ cells detected in highly inflammatory environments may be functionally unreliable. Second, stability concerns exist: maintenance of the Treg suppressive phenotype requires sustained high *FOXP3* expression and epigenetic stability at the locus. Thymus-derived natural Tregs (tTregs) typically exhibit high demethylation in the Treg-specific demethylated region (TSDR) of the *FOXP3* promoter, thereby ensuring stable *FOXP3* expression (27, 28); whereas peripherally induced Tregs (pTregs/iTregs) display lower *FOXP3* gene demethylation and are thus prone to phenotypic drift. Studies indicate that iTregs are more susceptible to loss of *FOXP3* expression and conversion to proinflammatory T cells in inflammatory settings, whereas stable natural Tregs retain their phenotype even under inflammation. Therefore, measuring *FOXP3* expression levels alone cannot determine Treg origin or stability, necessitating more in-depth analyses such as TSDR methylation status. Several emerging markers have been employed to aid in assessing Treg stability—for example, Helios expression levels mentioned earlier and Nrp1 patterns in mice—yet no absolutely reliable marker exists in humans to distinguish natural from induced Tregs, warranting further investigation.

Beyond the inherent specificity and interpretive limitations of the markers themselves, differences in sample sources also influence the assessment of Treg phenotype and functional status. Peripheral blood and graft tissue reflect distinct immune microenvironments; consequently, Treg phenotypic characteristics and functional states may differ between the two compartments (29). Peripheral blood Tregs predominantly represent circulating populations and are typically used to characterize systemic immune regulation (29); whereas within the graft and its draining lymphoid organs, Tregs are more likely to exhibit activated or tissue-adapted phenotypes, with their proportions and suppressive functions being more profoundly influenced by local inflammation levels, cytokine profiles, and metabolic conditions (30). Therefore, Treg-related indices in peripheral blood do not necessarily align with the local immune status within the graft.

In the context of heart transplantation, the definition and assessment of Tregs must contend with limitations in both marker interpretation and sample source selection. *FOXP3* remains an indispensable core marker in Treg research. However, its limited specificity and inability to independently reflect cellular phenotypic stability or true suppressive function render it more suitable as a component of multimarker panels, ideally supplemented, when feasible, with epigenetic evidence of stability for additional constraint. Concurrently, peripheral blood, the graft, and its draining lymphoid organs reside in distinct immune microenvironments, such that Treg phenotype and functional status may differ between these compartments. Consequently, peripheral blood indices should not be regarded as direct surrogates for local graft immune status.

## 4 Early post-transplant phase (0–30 days): Treg dynamics in tissue injury and inflammation

The first 0–30 days after heart transplantation constitute the perioperative and early recovery period. During this phase, the graft undergoes intense non-specific injury stimuli, including surgical trauma and ischemia-reperfusion injury (IRI), triggering acute inflammatory responses (8). IRI promotes the release of abundant damage-associated molecular patterns (DAMPs), such as high-mobility group box 1 (HMGB1) and fibronectin, thereby activating innate immune cells and driving their massive infiltration into the graft (31). These innate immune responses not only directly cause tissue damage but also amplify cascade effects through the release of proinflammatory cytokines and chemokines, laying the foundation for subsequent adaptive immunity (8). Clinically, intensified immunosuppression initiated immediately post-transplant reaches peak intensity during this period. This period is frequently characterized by discordance between local and systemic immune status: the graft locally features predominantly sterile inflammation and repair responses, whereas peripheral immune reactions are markedly suppressed by immunosuppressive therapy (32). As regulators of adaptive immunity, Tregs may experience concurrent influences on their numbers and function from local inflammatory signals and systemic pharmacologic effects during this stage.

### 4.1 Impact of induction therapy and early immunosuppression on Tregs

Many heart transplant recipients receive induction immunosuppression intraoperatively or in the early postoperative period, such as antithymocyte globulin (ATG) or the anti-IL-2 receptor antibody basiliximab (33). Although these agents effectively attenuate initial T cell responses, they also exert effects on Tregs (34). ATG, a polyclonal T cell-depleting antibody, non-selectively depletes T lymphocytes, including Tregs, resulting in a substantial decline in peripheral T cell counts during the early postoperative phase; whereas basiliximab, an anti-CD25 monoclonal antibody, blocks IL-2 signaling—a factor essential for Treg survival—and may thereby transiently restrict Treg proliferation and maintenance (35). Consequently, peripheral blood Treg counts in recipients are frequently markedly lower than preoperative baselines during the first month post-transplant and require time to recover (36). For example, in pediatric heart transplant patients undergoing thymectomy, the absence of thymic neogenesis of T cells combined with immunosuppressive effects leads to a progressive decline in peripheral Treg proportions starting from the seventh month postoperatively (37). Although thymectomy is not routine in adult patients, high-dose corticosteroids and calcineurin inhibitors (CNIs) during the perioperative period similarly suppress Tregs (38). Some studies indicate that calcineurin inhibitors indirectly reduce Treg expansion by suppressing IL-2 production, whereas rapamycin is relatively sparing of Treg survival (39). In a rat heart transplant model, Huang et al. found that single-dose rapamycin upregulated *FOXP3* expression in spleen and lymph nodes but did not significantly alter peripheral blood Treg proportions (40). This observation highlights that peripheral blood Treg counts do not always align with Treg changes in lymphoid tissues, suggesting potential informational gaps when using blood metrics to reflect rejection-related immune processes. Overall, early intensified immunosuppression frequently places circulating Tregs in a nadir or functionally suppressed state, potentially diminishing their capacity to exert regulatory effects during the initial transplant phase.

### 4.2 Tregs in the ischemia-reperfusion injury (IRI) milieu

The robust innate immune activation triggered by IRI not only directly damages cardiomyocytes but also alters the local immunoregulatory microenvironment. In the early post-transplant period, IRI and tissue stress trigger sterile inflammation and endothelial activation, inducing chemokine release and upregulation of adhesion molecules, thereby promoting immune cell migration from circulation to the graft and its draining lymph nodes, with subsequent local accumulation. Subsequently, alloantigen-driven adaptive immune responses can further amplify inflammation and modify the local microenvironment (41). Post-homing Treg retention, stability, and suppressive function are continually regulated by factors such as cytokine profiles and metabolic conditions (23, 42). Numerous studies demonstrate that certain innate immune signaling pathways affect Treg function: for example, activation of Toll-like receptor (TLR) signaling by pathogen-associated molecular patterns (PAMPs) impairs Treg suppressive capacity, while complement components C3a/C5a can also modulate Treg activity via their receptors (43). Recent studies have revealed that activation of the thrombin-protease-activated receptor-1 (PAR-1) pathway during IRI promotes inflammatory cascades via downstream inflammasomes and proinflammatory cytokines, potentially suppressing Treg suppressive function and impairing local immune tolerance (44).

Furthermore, in highly inflammatory environments, Tregs exposed to elevated proinflammatory cytokines such as IL-6 may undergo phenotypic and functional alterations, potentially transdifferentiating into phenotypes that secrete proinflammatory cytokines like IL-17 (45). In other words, proinflammatory mediator release and innate immune activation driven by tissue injury in the early transplant phase may diminish Treg suppressive function, thereby attenuating control over effector T cell responses and limiting their role in rejection suppression. In this context, attenuating innate immune responses associated with IRI may create a more favorable microenvironment for Treg-mediated immunoregulation. Supporting evidence comes from animal studies: in a mouse heart transplant model, perfusion of the donor heart with the membrane-targeted thrombin inhibitor Thrombalexin (TLN) to mitigate IRI reduced proinflammatory chemokine levels in the recipient graft, while increasing infiltration by anti-inflammatory phenotype macrophages and Tregs. Combined administration of TLN and donor-specific Treg infusion significantly prolonged graft survival, outperforming Treg infusion alone (46). Thrombin inhibition alleviated early inflammation-mediated leukocyte chemotaxis, fostering a microenvironment more conducive to Treg recruitment and function. This evidence suggests that, in the early transplant phase, controlling excessive innate immune activation or supplementing anti-inflammatory factors could enhance the regulatory efficacy of endogenous or exogenous Tregs against acute injury and immune responses.

### 4.3 Treg recruitment and function in the early graft

Although peripheral Tregs are suppressed during the early phase, the host may still mobilize Tregs to damaged tissues to control inflammation (47). Animal studies indicate that early Treg infiltration in organ IRI models positively correlates with tissue repair and damage control: for instance, in mouse models of renal, cardiac, and hepatic ischemia-reperfusion, Tregs entering injured sites early help mitigate tissue necrosis and promote functional recovery (48). Tregs may suppress excessive activation of neutrophils and macrophages via pathways involving IL-10 and TGF-β, thereby limiting the spread of inflammation (49). Additionally, Tregs can release growth factors such as amphiregulin (AREG) in damaged myocardium, promoting cardiac fibroblast proliferation and angiogenesis, thus accelerating tissue repair (50). Notably, this tissue repair-promoting function of Tregs represents another important aspect distinct from their conventional immunosuppressive role. In acute tissue injury settings, a subset of Tregs expresses the IL-33 receptor ST2; upon binding injury-released IL-33, these cells upregulate and secrete AREG and IL-13, thereby activating tissue fibroblasts and inducing tissue macrophages toward a pro-repair phenotype (51). In acute tissue injury environments, IL-33, as an alarmin, is upregulated in damaged endocardial endothelial cells and cardiomyocytes. The ST2+ subset of receptor-bearing Tregs, upon IL-33 stimulation, locally secretes AREG and IL-13, thereby both suppressing immune responses and promoting myocardial tissue reconstruction (52). Thus, in the early post-transplant period, Treg entry into the graft may exert dual effects: suppressing excessive immune-mediated injury on one hand and promoting repair of damaged myocardium on the other. However, clinical observations of Tregs in early heart transplantation reveal a more complex picture. In most patients with adequate immunosuppression, overt acute T cell-mediated rejection is rare in the initial weeks post-transplant, during which lymphocyte infiltration in graft tissue is relatively limited and Treg numbers remain low (53). In contrast, in patients experiencing early rejection, Tregs frequently appear in large numbers in the graft as responsive cells.

**Figure 1:**
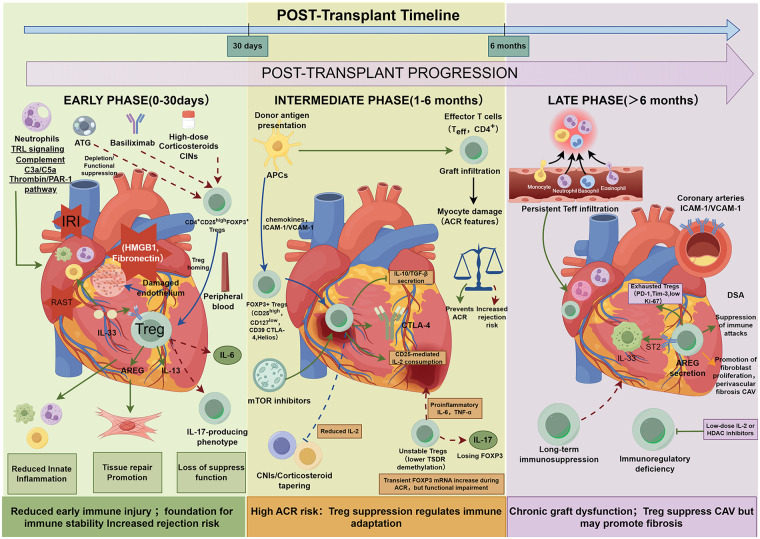
Schematic overview of regulatory T cell (Treg) dynamics in three stages following heart transplantation, structured along a horizontal timeline with panels for the early phase (0–30 days), intermediate phase (1–6 months), and late phase (>6 months). Each panel features a stylized heart graft illustrating stage-specific immune processes. In the early phase, ischemia-reperfusion injury releases damage-associated molecular patterns activating innate immunity; induction immunosuppression suppresses Tregs; recruited Tregs activated by IL-33/ST2 secrete AREG/IL-13 for dual inflammation suppression and tissue repair, though proinflammatory cytokines like IL-6 induce instability leading to IL-17 production, reducing early injury or increasing rejection risk. The intermediate phase shows donor antigen activating effector T cells causing graft infiltration and myocyte damage (acute cellular rejection); Tregs recover and suppress via IL-10/TGF-β, CTLA-4, and IL-2 consumption; mTOR inhibitors support stability, calcineurin inhibitors impair it; proinflammatory cytokines drive instability to IL-17, with transient *FOXP3* mRNA rises during rejection, balancing high rejection risk and immune adaptation. The late phase depicts chronic inflammation, ICAM-1/VCAM-1 upregulation, and DSA promoting cardiac allograft vasculopathy; exhausted Tregs suppress attacks but exhibit bidirectional IL-33/ST2/AREG effects promoting suppression alongside fibrosis; long-term immunosuppression reduces Tregs, causing immunoregulatory deficiency and graft dysfunction, potentially mitigated by low-dose IL-2 or HDAC inhibitors.

Overall, the role of Tregs in the 0–30 day post-transplant phase depends on the interplay between local injury-related inflammation intensity and systemic immunosuppression. On one hand, ischemia-reperfusion injury and associated innate immune activation can impair Treg suppressive function, while postoperative immunosuppressive regimens may also constrain Treg numbers and functional expression; on the other hand, when anti-donor immune responses intensify, Tregs can be recruited and increase within the graft. Elevated *FOXP3*-related signals do not necessarily indicate tolerance but more likely reflect negative immunoregulatory attempts in an inflammatory context, with outcomes depending on whether Tregs maintain their suppressive phenotype and function. When rejection intensity is high or Treg function is compromised, increases in endogenous Tregs are often insufficient to prevent tissue damage. Based on available animal studies, interventions in the early phase can be summarized along two paths: reducing injury-related inflammation and innate immune responses to improve the microenvironment for Treg function, while enhancing Treg recruitment and expansion under controlled safety conditions to augment local suppressive capacity. Thus, Treg quantity and functional status during the 0–30 day phase should be regarded as critical early determinants influencing subsequent immune trajectories, with effective modulation potentially reducing acute immune injury and establishing a foundation for later immune stability (Figure 1).

## 5 Intermediate post-transplant phase (1–6 months): Treg dynamics and role during the high-risk period for acute rejection

The 1–6 month period post-transplantation is regarded as the phase of acute immune adaptation. During this interval, patients gradually recover from the perioperative period, and various immune cell subsets begin to reestablish equilibrium following induction and intensified immunosuppression (54). Clinically, this intermediate phase represents the peak period for acute cellular rejection (ACR): the majority of ACR episodes in heart transplant recipients occur within the first 3 months, with a marked decline after 6 months (55). This pattern relates to postoperative adjustments in immunosuppressant concentrations and the gradual development of adaptive immune responses to the allograft. Within the first 1–3 months, corticosteroid doses are typically tapered and calcineurin inhibitor levels adjusted to minimize drug toxicity; concurrently, patients recover from surgery and complications, with lymphocyte function rebounding. In this context, T cell responses to donor antigens may intensify, potentially leading to rejection if immunosuppression is inadequate (9). Tregs are considered a key factor influencing rejection risk during this intermediate phase: their abundance and functional potency largely determine whether the host can balance aggressive immune responses against the graft (54). In this phase, donor-specific effector T cell responses progressively strengthen, and Treg suppressive capacity determines the direction of immune balance: dominance of effector responses favors graft injury and acute rejection, whereas effective Treg restraint of effector activity promotes graft stability (36).

### 5.1 Peripheral Treg counts and proportions in the intermediate phase

Following transplantation, Treg counts, which decline early due to induction therapy, partially recover over weeks to months, although the extent and sustainability of this recovery vary among individuals (56). In adult patients, thymic involution after maturity limits peripheral T cell repertoire replenishment primarily to survival and homeostatic turnover, constraining Treg reconstitution rates (57). Pediatric patients theoretically retain stronger thymic function; however, many undergo thymectomy during cardiac transplantation, thereby restricting postoperative Treg sources. A follow-up study in pediatric heart transplant recipients demonstrated a progressive decline in peripheral blood Treg proportions starting from the seventh postoperative month, remaining persistently low thereafter (58). Lopez-Abente et al., reported that this Treg reduction correlates with increased rejection risk, suggesting that failure to restore Tregs beyond 6 months may lead to immunoregulatory imbalance (34). Adult studies similarly observe relatively higher Treg proportions in stable recipients several months post-transplant, whereas those experiencing rejection often exhibit lower proportions (56). This pattern is also reported in studies of kidney transplant operational tolerance and meta-analyses: higher Treg levels in recipients generally associate with improved graft function and reduced acute/chronic rejection (59). Particularly in high-risk recipients, maintaining higher Treg frequencies is often associated with lower rejection rates. Therefore, maintenance of peripheral Treg counts during the 1–6 month phase is regarded as a key indicator of transplant immune balance.

### 5.2 Relationship between Tregs and ACR

Acute cellular rejection is an attack response mediated by recipient T lymphocytes, characterized pathologically by interstitial lymphocytic infiltration in the myocardium accompanied by myofiber damage (60). As suppressors within the T cell lineage, Tregs exhibit a complex inverse relationship with ACR. Substantial evidence indicates that Treg deficiency or impaired function increases the risk of ACR. For example, studies comparing immune profiles between patients experiencing ACR and those without rejection have found markedly reduced suppressive function of peripheral Tregs in the former group. Specifically, although peripheral *FOXP3*+ T cell counts in ACR patients may not be significantly reduced, their phenotype more closely resembles effector T cells—for instance, upregulated CD127 expression and inadequate expression of inhibitory molecules such as CTLA-4—indicating functional defects (61). These Tregs also perform poorly in *in vitro* suppression assays, failing to effectively inhibit proliferation of cognate effector T cells. E. Dijke et al. analyzed endomyocardial biopsies from heart transplant recipients and found that found that FOXP3 mRNA expression levels in biopsy tissues during ACR were significantly higher than in rejection-free periods. This upregulation likely reflects a compensatory feedback loop triggered by immune activation, rather than an effective establishment of tolerance (26, 62). This finding contrasts with expectations, as *FOXP3*+ cells are typically regarded as suppressive, with their increased numbers generally associated with reduced rejection. However, the authors noted that *FOXP3* upregulation reflects activation of immunoregulatory mechanisms, which depends on the presence of an ongoing immune response.

In other words, robust anti-donor immune responses can coincide with Treg recruitment and expansion, more likely reflecting the host’s initiation of negative immunoregulatory processes amid immune activation. Further evidence supporting this view is that functional Tregs can be expanded from *ex vivo* cultures of cardiac biopsies during ACR. In 5 heart transplant recipients with ACR, *ex vivo* culture of infiltrating lymphocytes from endomyocardial biopsies for 18–21 days yielded approximately 0.5–1.0 × 10^6^ viable cells per case, with a median of about 10.6% being *FOXP3*+ T cells, indicating the presence of an expandable regulatory T cell population among ACR-infiltrating lymphocytes (63). These results demonstrate that, even in the inflammatory environment of ongoing rejection, a certain number of Tregs persist within the graft and can proliferate under appropriate conditions. Nevertheless, although Tregs are recruited during acute rejection, they may not suffice to curb the ongoing rejection episode. In subsequent studies, Baan et al. used *FOXP3* gene TSDR demethylation analysis to show that *FOXP3*+ cells infiltrating the cardiac graft during acute rejection are predominantly thymus-derived natural Tregs, confirming that host natural Tregs migrate into the graft to participate in the immune response (64). However, these natural Tregs failed to prevent the ultimate occurrence of rejection. Conversely, evidence suggests that their numbers may transiently increase before rejection onset, followed by a decline: Peyster et al.’s *in situ* immune analysis of endomyocardial biopsies revealed dynamic changes in *FOXP3*+ cell infiltration levels, with a detectable trend toward reduced *FOXP3*+ cell proportions prior to clinical rejection. In other words, when local Tregs are insufficient, rejection propensity may rapidly progress to substantive rejection (65).

### 5.3 Local Treg responses in the graft during the intermediate phase

Heart transplant patients undergo multiple endomyocardial biopsies at 1, 2, 3, and 6 months post-transplant to monitor for asymptomatic rejection (66). Immunohistochemical and gene expression analyses of these biopsy samples reveal the dynamics of Tregs within the graft. Generally, in stable patients without rejection, biopsies show sparse *FOXP3*+ lymphocytes in the myocardial interstitium, albeit in low numbers (25). In contrast, during subclinical or mild rejection, *FOXP3*+ cell numbers increase, commonly near inflammatory foci, suggesting Treg recruitment in an attempt to control local immune activation (67). This recruitment is typically associated with rejection-related local inflammation, where inflammatory foci generate chemotactic signals and upregulate endothelial adhesion molecules, thereby enhancing the probability of Treg entry into the graft and accumulation around lesions (68). When rejection reaches moderate or higher grades (2R), *FOXP3*+ cells often accumulate markedly. Peyster et al., reported that in some patients, *FOXP3*+ cell peaks precede definitive histologic rejection diagnosis, with subsequent density decline as rejection progresses. This may imply that Tregs initially attempt to suppress rejection, but if rejection is not promptly controlled, persistent inflammation could lead to Treg exhaustion or egress, resulting in pathologically observed reductions in *FOXP3*+ cells, followed closely by severe tissue destruction. This inference requires validation through additional serial biopsy studies but conceptually aligns with prior observations that reduced *FOXP3*+ cells may signal heightened risk of subsequent rejection (65).

Therefore, a gradual decrease in Treg infiltration within the graft or an imbalance in the Treg/Teff (69) (effector T cell) ratio detected during the intermediate phase may signal elevated rejection risk, necessitating intensified immunosuppression or alternative interventions. Clinical studies using endomyocardial biopsies have employed *FOXP3* TSDR demethylation to distinguish natural Tregs and found that these cells can infiltrate the graft prior to rejection yet fail to prevent subsequent episodes, while overall Treg levels negatively correlate with the number of rejection events in the first year. This underscores the need to concurrently monitor Treg subset stability, functional status, and dynamic changes rather than relying solely on single time-point counts.

### 5.4 Immunosuppressive drug adjustments and Treg balance

During the 1–6 month period, clinicians frequently adjust immunosuppressive regimens based on patient status, and these modifications also affect Treg numbers and function (70). A common strategy involves gradual tapering of corticosteroid doses in the early postoperative phase, as prolonged high-dose corticosteroids increase risks of infection and metabolic complications. However, corticosteroids also non-specifically inhibit T cell proliferation, including Tregs, and whether rapid dose reduction benefits Tregs remains unclear (71). Calcineurin inhibitors are typically maintained at higher blood concentrations during this intermediate phase to prevent rejection, but they restrict T cell proliferation by suppressing IL-2 transcription, thereby also impairing Treg utilization of IL-2 (72). This may partially offset Treg count recovery. Consequently, some centers attempt alternative agents in the mid- to long-term, such as mTOR inhibitors (73), to replace CNIs or reduce their doses. mTOR inhibitors are considered more Treg-friendly, as they inhibit effector T cell proliferation while relatively preserving or even promoting Treg expansion (74). Clinical studies show that early conversion to sirolimus in heart transplant patients is associated with lower ACR incidence and slowed progression of cardiac allograft vasculopathy (CAV) during long-term follow-up (75). Although multiple factors are involved, Treg contribution may play a role: under rapamycin exposure, patient blood Treg proportions increase, favoring maintenance of graft immune tolerance (76). Overall, adjustments to immunosuppressive strategies in the intermediate phase must balance effects on effector and regulatory immunity, ideally preventing rejection without substantially impairing Treg-mediated regulatory function.

Treg suppressive functional status is equally critical during the intermediate phase. At this time, persistent donor antigen stimulation keeps most Tregs in an activated state. If Tregs maintain a stable suppressive phenotype, they can effectively suppress effector T cells and induce exhaustion or an exhausted phenotype in some effector cells (69, 77, 78). Conversely, loss of Treg stability may lead to immune imbalance. One manifestation of Treg instability is the production of proinflammatory cytokines (79). In human peripheral blood, stable Tregs generally constitute a high proportion, with most *FOXP3*+ cells resisting phenotypic conversion absent extreme inflammation. However, in rejection settings, locally elevated concentrations of IL-6 and TNF-α may induce unstable Tregs to produce IL-17, thereby attenuating overall suppressive efficacy (80). Clinical cytokine profiling of ACR biopsies reveals elevated Th17-related factors such as IL-17 alongside classical Th1 cytokines in rejection tissues, suggesting a potential Treg/Th17 balance shift (73). Therefore, in the management and research design for the intermediate transplant phase, some studies propose enhancing Treg stability and suppressive function as a potential intervention target. For example, ICEP-type small molecules or signaling pathway interventions can enhance *FOXP3* acetylation levels and suppress inflammatory signal interference, thereby locking in Treg suppressive function (81, 82). This approach remains in the research stage, but its rationale is to further bolster Treg-mediated immunoregulation on top of standard immunosuppression. Additionally, certain physiologic factors such as type I interferons can, under specific conditions, enhance Treg stability, purportedly by promoting *FOXP3* acetylation (83). This suggests that finer tuning of immunosuppressant combinations during the intermediate phase could favor Treg maintenance. For instance, moderate use of tamoxifen or IFN-β therapy may help augment Treg contributions to rejection resistance, warranting further validation.

The 1–6 month period post-transplantation represents a phase of high acute rejection risk coupled with gradual adjustment of immunosuppression. During this phase, Tregs progressively recover from the nadir induced by induction therapy, with their levels and suppressive functional status in peripheral blood and the graft correlating with ACR risk. Tregs contribute to regulating alloimmune responses through mechanisms including suppression of effector T cell activity, modulation of antigen-presenting cell function, and alteration of IL-2 availability. Immunosuppressive regimens influence Treg maintenance and function; thus, management during this phase requires balancing rejection risk reduction with preservation of immunoregulatory capacity, while providing a foundation for subsequent translational research (Figure 1).

## 6 Late post-transplant phase (>6 months): Treg role in chronic inflammation and vasculopathy

In the late post-transplant period, from 6 months to several years or longer, the incidence of acute rejection markedly decreases, but chronic immune-mediated injury progressively accumulates, manifesting as chronic graft dysfunction and characteristic CAV (84). CAV is a diffuse process of coronary intimal proliferation and narrowing, considered the result of donor-specific immune responses interacting with persistent low-grade inflammation, endothelial injury, and smooth muscle proliferation (85). Patients in this phase are typically on maintenance immunosuppression, with drug doses reduced compared to the early period to balance long-term drug toxicity against rejection risk. However, prolonged use of CNIs and other immunosuppressants may itself impair regulatory immune capacity (70). As Riella noted, most currently used immunosuppressive drugs exert negative effects on the immunoregulatory arm, contributing in part to suboptimal long-term survival rates (38). Consequently, in the late phase beyond 6 months, Treg numbers are frequently below normal physiologic levels, with potential functional impairment. Clinical observations support this: a pediatric study found persistent reduction in peripheral Treg frequencies after 6 months post-heart transplant, becoming significantly lower than normal reference ranges by 1 year (37). Bernaldo de Quiros et al. further emphasized that this Treg depletion significantly correlates with subsequent acute rejection and immune dysregulation. In adult experience, heart transplant recipients maintained on long-term regimens combining tacrolimus, mycophenolate mofetil, and low-dose prednisone exhibit chronic immunosuppressive effects, including reduced total T cell counts, predominance of memory phenotypes, and limited Treg proliferation due to prolonged dependence on exogenous IL-2 (58).

The late phase can be viewed as a state of immunoregulatory deficiency amid chronic inflammation: systemic immune responses are controlled at low levels, yet regulatory immune recovery remains poor, with persistent local chronic inflammatory stimuli driving the development of CAV and other chronic lesions.

### 6.1 Tregs and CAV

CAV represents one of the primary factors affecting long-term prognosis in heart transplantation. By 10 years post-transplant, approximately half of recipients develop CAV, underscoring its high cumulative burden in the late phase (86). The pathogenesis of CAV is complex, involving both immune and non-immune factors, with chronic immune rejection serving as a key driver that leads to coronary endothelial inflammation, smooth muscle cell migration and proliferation, and collagen deposition (85). As negative regulators of immune responses, Tregs are theoretically protective against chronic rejection and CAV. Sustained Treg activity in the long term could mitigate chronic vascular injury by suppressing persistent effector T cells within the graft, thereby reducing immune attacks on endothelial cells, and by inhibiting helper T cell assistance to B cells, thus limiting donor-specific antibody production (87). Indeed, studies in tolerant animal models show that coronary arteries in long-surviving grafts exhibit minimal CAV, whereas Treg depletion markedly exacerbates CAV (88). Additionally, clinical observations in rare kidney or liver transplant patients who achieve long-term tolerance after immunosuppression withdrawal reveal higher peripheral Treg levels compared to those on conventional therapy, with virtually no chronic rejection lesions in these individuals. These findings indirectly support an inhibitory role of Tregs in chronic graft injury (89).

Beyond T-cell regulation, the interaction between Tregs and the humoral immune arm is critical in the late phase. T follicular regulatory (Tfr) cells are specialized subsets that suppress T follicular helper (Tfh) cells, thereby limiting B-cell help and subsequent Donor-Specific Antibody (DSA) production. A deficiency or dysfunction in the Tfr/Tfh balance has been linked to the development of chronic antibody-mediated rejection and CAV.

However, recent studies suggest that Tregs may exert bidirectional effects in the late post-transplant period. Beyond immunosuppression, Tregs also participate in tissue repair and remodeling processes (52). In lung transplantation studies, Treg-derived AREG promotes hyaluronan production by bronchial epithelium, and excessive hyaluronan accumulation is associated with chronic lung graft dysfunction (90). The Turnquist team demonstrated in a mouse heart transplant model that IL-33/ST2 axis-driven Treg responses in the chronic phase may promote fibrosis and vascular remodeling, thereby exacerbating processes related to chronic allograft vasculopathy. In an accelerated chronic rejection model, elevated donor heart IL-33 expression rendered graft-infiltrating Tregs the primary source of AREG, accompanied by fibroblast proliferation and increased perivascular fibrosis; conversely, in recipients lacking *AREG*, these fibrotic changes and T cell infiltration were both attenuated (91). This study indicates that Treg-mediated tissue repair effects may transform into profibrotic actions in the context of chronic inflammation, necessitating attention to their potential impact on tissue remodeling alongside maintenance of immunosuppression. Accordingly, the IL-33/ST2/AREG pathway has been proposed as a potential regulatory target, although its translational value and effects on rejection require further evaluation.

### 6.2 Tregs and the chronic inflammatory microenvironment

In the late post-transplant myocardium, low-grade chronic inflammation is common, characterized by scattered infiltration of monocytes, macrophages, and T cells, along with persistently elevated endothelial expression of adhesion molecules such as ICAM-1 and VCAM-1 (92). Persistent upregulation of adhesion molecules, in conjunction with local chemokine signals, facilitates the entry of T cells—including Tregs—from circulation into the graft and their enrichment around perivascular lesions, resulting in long-term coexistence of immunoregulation and inflammatory injury in the chronic phase (93). This chronic inflammation is driven on one hand by incompletely cleared immune effectors and on the other by non-immune factors such as transplant arteriosclerosis and recurrent minor injuries (94). In such microenvironments, Tregs typically exist in a state of chronic activation or exhaustion. Pathologic studies of endomyocardial biopsies associated with chronic rejection reveal *FOXP3*+ Treg infiltration, often accompanied by elevated expression of exhaustion-related molecules such as PD-1 and Tim-3, alongside reduced Ki-67 proliferation levels, suggesting functional limitation in this population (29). An analysis of long-term transplant survivors found that peripheral Tregs in chronically stable patients maintain higher levels of CTLA-4 and IL-10 production, whereas Tregs in patients with progressive CAV exhibit reduced expression of these functional markers. This may indicate that Tregs in the former group are functionally more intact, contributing to suppression of chronic inflammation progression (95). Additional studies show that in CAV mouse models, *Nox2* gene deletion in Tregs enhances their suppressive function and migration capacity, thereby alleviating cardiac transplant vasculopathy. Distinctly, another study demonstrated that Areg-deficient Tregs exhibit improved survival and graft infiltration, resulting in reduced arterial narrowing in the graft (96). This suggests that modulating metabolism and the microenvironment can strengthen Treg function in the chronic phase, enabling more effective control of inflammation and lesion progression.

### 6.3 Long-term Treg maintenance and immune monitoring

Given the adverse effects of long-term immunosuppression on Tregs, maintaining sufficient Treg function in the late phase represents a critical challenge (97). On one hand, optimization of immunosuppressive regimens is required, for example by gradually reducing doses of agents with pronounced Treg-suppressive effects or selecting Treg-sparing drug combinations, to maximally preserve Treg numbers and function and thereby facilitate the establishment of immune tolerance (98). If partial reduction or even withdrawal of immunosuppressants can be achieved while supporting immune tolerance through alternative measures, Treg reconstitution may become feasible. Preliminary human clinical trials have shown that low-dose IL-2 safely promotes circulating Treg expansion in settings such as liver transplantation; however, monotherapy with low-dose IL-2 has not significantly induced graft tolerance or permitted withdrawal of conventional immunosuppression, indicating the need for future combination with other immunoregulatory strategies to improve efficacy (99). On the other hand, immune monitoring in the chronic phase requires the introduction of novel markers. Existing studies indicate that expression levels of the Treg marker *FOXP3* and the immunoregulatory cytokine IL-35 in peripheral blood of organ transplant recipients correlate with long-term graft function and rejection events: higher *FOXP3* and IL-35 expression is typically associated with favorable transplant outcomes, whereas lower levels correspond to poorer graft function and heightened immune activity (100). This demonstrates that high Treg activity levels are associated with superior long-term prognosis. Therefore, incorporating select Treg functional markers into late-phase follow-up could serve as indicators of immune balance deviation. Detection of declining Treg-related markers may signal the need for treatment adjustments to restore immunoregulation.

In the late post-transplant phase, Treg numbers typically decline under conventional conditions, accompanied by reduced immunoregulatory capacity. This creates opportunities for chronic low-grade immune attacks, driving the development of CAV and fibrotic lesions. Adequate and stable Tregs hold promise for protecting the graft by suppressing chronic immune responses; conversely, insufficient numbers or dysfunctional Tregs render chronic rejection difficult to control. Notably, Tregs in the chronic phase are not unequivocally beneficial, as their tissue repair-promoting functions may exacerbate fibrosis under specific pathways. Future interventions must balance Treg suppressive and reparative roles or target specific molecules such as IL-33/AREG for modulation. Overall, sustaining adequate Treg numbers and function in the late phase is key to prolonging graft survival, requiring optimization of immunosuppressive regimens and incorporation of novel immunotherapeutic approaches (Figure 1).

## 7 Distribution differences of Tregs between peripheral blood and the graft

In routine follow-up of transplant recipients, peripheral blood samples represent one of the primary and most practical sources for obtaining immune information. Clinical and research practices commonly employ flow cytometry to analyze immune cell subset proportions and phenotypes, measure circulating cytokine levels, and monitor donor-specific antibody (DSA) titers to infer recipient immune status and graft function (101). However, increasing evidence indicates that peripheral blood immune markers may not adequately reflect ongoing immune processes within the graft. Detected immune cell subsets, cytokines, or Treg quantities and phenotypes in peripheral blood frequently diverge from the local immune status inside the graft, and sole reliance on these peripheral blood indices risks misjudging graft rejection or tolerance states (102).

### 7.1 Treg homing and distribution differences

Transplant immune responses involve multiple anatomical compartments, including secondary lymphoid organs, peripheral blood circulation, and graft tissue, where relevant immune cells migrate between these compartments and collectively determine local and systemic immune status. Tregs exhibit dynamic migratory capacity under both homeostatic and inflammatory conditions; under donor antigen stimulation, a subset of Tregs can traffic from circulation into secondary lymphoid organs to directly interact with antigen-presenting cells and effector T cells, whereas another subset undergoes chemokine-guided directional migration into the graft to exert suppressive function, thereby coordinating immune tolerance and rejection responses (103). Consequently, Tregs in blood represent only a fraction of the total body Treg pool, with their numbers heavily influenced by distribution factors. Studies in rodent allogeneic heart transplant models have shown that rapamycin treatment significantly increases *FOXP3*+ Treg numbers and *FOXP3* expression levels in recipient spleen and peripheral lymph nodes, without notable changes in peripheral blood Treg counts, indicating that the effects of immunosuppressants on Treg dynamics in local lymphoid organs may not be directly reflected in peripheral blood (40). In other words, Tregs may undergo directional migration toward graft tissue and exert effects locally, resulting in minimal observable changes in their peripheral blood numbers.

Clinical and experimental studies have observed that when local immune responses occur within the graft, Tregs can be recruited by chemokines and antigen signals from inflammatory sites to infiltrate the graft and its draining lymph nodes for immunoregulation. This may result in decreased or unchanged Treg proportions in peripheral blood, which does not indicate diminished overall immunoregulatory capacity, as tissue-resident Tregs are actively engaged in countering rejection (104). It should be emphasized that Treg homing or elevated *FOXP3* signaling *per se* does not equate to immune tolerance, as these events frequently occur against the backdrop of activated local immune responses. The significance of homing depends on whether Tregs maintain stable suppressive function locally and their relative balance with effector T cell response intensity; thus, interpretation should integrate tissue inflammation degree with other immune markers (103).

### 7.2 Impact of the local microenvironment on Tregs

The immune microenvironment within graft tissue differs markedly from that in peripheral blood. The composition, activation status, and cell-cell interaction patterns of immune cells in local tissue frequently diverge from those in circulating blood, as revealed by multiple transcriptomic and single-cell analyses (105). Therefore, interpreting peripheral blood immune markers requires caution and should incorporate graft microenvironmental features to achieve a comprehensive understanding of transplant immune responses (105). Upon entering the graft, Treg phenotype and function are modulated by the local environment, resulting in differences from peripheral blood Tregs. The graft internal immune microenvironment is typically replete with high levels of proinflammatory cytokines and stress signals associated with tissue injury and ischemia-reperfusion. These factors not only serve as key mediators of rejection but also generate local hypoxia, metabolic stress, and signaling pathway activation that can profoundly alter gene expression and functional status in infiltrating immune cells. Existing studies show that local proinflammatory cytokine levels are markedly elevated during the acute transplant phase, and hypoxia along with other microenvironmental signals can directly influence Treg differentiation and function-related gene expression profiles via transcription factor regulation. Consequently, Treg gene expression in the graft may differ substantially from that in peripheral blood, necessitating integration of local microenvironmental features for a holistic understanding of their regulatory role (106).

Single-cell RNA-seq studies have clearly demonstrated substantial differences in transcriptional profiles and differentiation trajectories between circulating Tregs and tissue-infiltrating/tissue-resident Tregs, with circulating Tregs exhibiting gene expression patterns more akin to a naïve-to-memory transition, whereas tissue Tregs display features highly adapted to the local microenvironment, including upregulation of chemokine receptors, integrins, and other molecules involved in tissue residency and cell-cell interactions. Even when Treg populations of comparable size are present in peripheral blood and tissue, their molecular characteristics and potential functions may differ profoundly, highlighting the need to incorporate tissue-level transcriptomic data in transplant immune monitoring for a comprehensive understanding of Treg roles (30). Therefore, peripheral blood Treg counts alone cannot infer whether tissue Tregs are exerting normal suppressive function. If peripheral blood Treg proportions are normal but these Tregs fail to successfully home to the graft, local graft immunity may still become dysregulated. Conversely, low peripheral blood Treg levels may be sufficient for maintaining local tolerance if most Tregs have deeply infiltrated the tissue and are highly activated. In summary, the tissue microenvironment shapes Treg function, information that blood tests cannot capture. The stage-specific characteristics of Tregs and the corresponding clinical monitoring recommendations are summarized in Table 1.

**Table 1:** Stage-specific characteristics of Tregs and clinical monitoring recommendations.

Stage	Dominant immune events	Immunosuppression context	Treg status & phenotype (blood vs. graft)	Clinical monitoring & actionable goals
Early phase (0–30 Days)	**IRI & Sterile Inflammation:** DAMPs release (e.g., HMGB1); TLR signaling.	**Induction:** T-cell depletion (ATG) or CD25 blockade (Basiliximab).	**Blood:** Severe depletion; counts usually below baseline.	**Goal:** Monitor reconstitution kinetics.
	**Innate Immunity**: Macrophage/Neutrophil activation.	**High-dose Steroids:** Non-specific suppression.	**Graft:** Recruitment for tissue repair (IL-33/ST2 axis); risk of inflammatory destabilization.	**Tools:** Routine flow cytometry (CD4/CD25/FOXP3).
				Note: Low counts are expected; focus on recovery trends.
Intermediate phase (1–6 Months)	**Peak ACR Risk:** Adaptive alloimmunity; Effector T cell (Teff) expansion.	**Maintenance Adjustment:** CNI tapering.	**Blood:** Gradual recovery; predominately memory phenotype.	**Goal:** Detect subclinical rejection; resolve blood-graft discordance.
	**Treg/Teff Imbalance:** Critical determinant of rejection.	**Drug Impact:** CNIs inhibit IL-2, limiting Treg expansion; mTORi preserves Tregs.	**Graft:** Discordance likely. High infiltration may signal active rejection (compensatory response) rather than tolerance.	**Tools:** Treg/Teff Ratio; Functional markers (e.g., CD127lo, CTLA-4).
				**Action:** Trigger biopsy if blood Treg/Teff ratio drops or inflammation rises.
Late phase (>6 Months)	**Chronic Injury (CAV):** Chronic low-grade inflammation.	**Long-term Maintenance:** Minimization strategies.	**Blood:** Stable or slow decline; risk of plasticity (Th17-like conversion).	**Goal:** Assess lineage stability & plasticity.
	**Humoral Immunity:** B-cell help/DSA production.	**Tolerance Induction:** Potential weaning in select cases.	**Graft:** Tissue-resident Tregs may promote fibrosis via Areg pathway.	**Tools:** Advanced Panel: TSDR demethylation; cytokine staining (IL-17 vs IL-10).
	**Fibrosis:** Tissue remodeling.			**Action:** Monitor DSA; evaluate switch to Treg-sparing agents (e.g., Sirolimus) if stability is low.

### 7.3 Limitations of traditional single-marker detection

In clinical transplant immune monitoring, assessment of peripheral immune status often focuses on easily quantifiable and standardized single parameters, such as flow cytometry measurement of the percentage of CD4^+^CD25^+^*FOXP3*^+^ Tregs among CD4^+^ T cells to evaluate recipient immunoregulatory status (107). However, such single-parameter approaches are incomplete, as Treg functional status is more critical than quantity alone. Reporting peripheral blood Treg proportions alone fails to capture their functional heterogeneity, as Tregs can vary substantially in expression of inhibitory molecules such as CTLA-4/IL-10, *FOXP3* gene TSDR demethylation stability, and inflammation-related phenotypes—differences that conventional peripheral blood assays often cannot distinguish (23). Additionally, substantial inter-patient variability in baseline Treg levels complicates threshold establishment. Consequently, previous attempts to predict rejection using blood Treg counts have often yielded inconsistent results. Animal experiments by Huang et al. and certain clinical reports suggest that blood Treg counts may not serve as robust predictors of rejection, necessitating more in-depth functional assessments (40). To overcome these limitations, emerging technologies are increasingly applied to peripheral blood immune monitoring in clinical and research settings, aiming to surpass traditional single-marker evaluations. For example, multiparameter flow cytometry and mass cytometry (CyTOF) enable simultaneous quantification of dozens of cell surface and functional markers, yielding high-dimensional immune cell phenotyping profiles; high-dimensional single-cell RNA sequencing resolves transcriptional profiles of individual immune cells, while T cell receptor (TCR) sequencing elucidates clonal structure and dynamics of adaptive immune responses. These technologies provide more comprehensive assessment of immune cell quantity, functional status, and clonal specificity, promising improved predictive value in immune monitoring (108).

Peripheral blood harbors multiple immune cell populations with complex interactions. Even if changes in Treg levels are detected, absence of integrated analysis with other immune parameters hinders accurate interpretation of their biological and clinical significance (109). Prior to rejection initiation, peripheral blood Tregs may decline, while effector T cells (Teffs) exhibit expansion or activation trends. Reporting Treg changes alone may overlook overall immune shifts driven by synchronous enhancement of the effector arm; similarly, relying solely on stable Treg levels may mask increased risk signaled by rising Teffs (58). Furthermore, other immune components, such as myeloid-derived suppressor cells (MDSCs), B cells, and NK cells, also play roles. Therefore, relying on blood Treg metrics alone is insufficient to capture the full picture.

**Figure 2:**
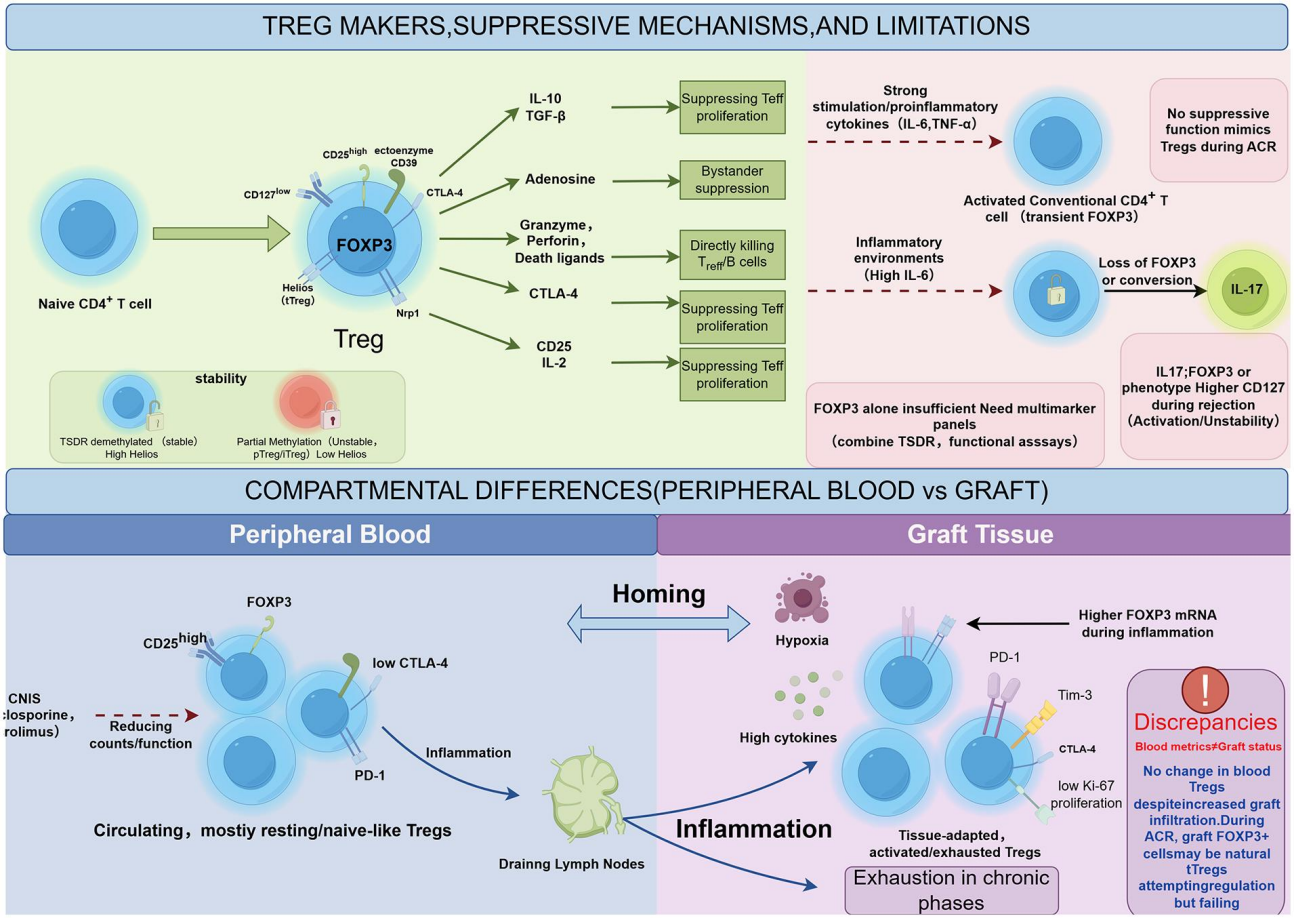
Schematic illustration of regulatory T cell (Treg) identification markers, suppressive mechanisms, detection limitations, and compartmental differences in heart transplantation. The figure is divided into upper and lower panels. The upper panel depicts Treg differentiation from naïve CD4+ T cells, centered on the *FOXP3* transcription factor, with surface markers including high CD25 expression, low CD127 expression, CD39, and CTLA-4; suppressive mechanisms include IL-10/TGF-β secretion to inhibit effector T cell proliferation, adenosine pathway for bystander suppression, granzyme/death ligands for direct killing of effector cells, CTLA-4 downregulation of costimulatory signals, and IL-2 consumption leading to effector T cell apoptosis; detection limitations are indicated by red dashed arrows, including proinflammatory cytokines like IL-6/TNF-α causing activated conventional T cells to transiently express *FOXP3* without suppressive function, unstable Tregs losing *FOXP3* or converting to IL-17-producing phenotype with low TSDR demethylation, emphasizing that single markers are insufficient and multimarker combinations are needed. The lower panel compares peripheral blood versus graft tissue compartments, connected by a homing arrow: peripheral blood Tregs are circulating with a resting/naïve-like phenotype, influenced by immunosuppression like CNIs; graft Tregs exhibit activated/tissue-adapted/exhausted phenotypes with PD-1, Tim-3, low Ki-67, influenced by local inflammation, hypoxia, high cytokines, and higher *FOXP3* mRNA during inflammation; discrepancies are highlighted, noting that blood indices do not reflect local graft status.

Peripheral blood markers cannot fully reflect immune processes within the graft for multiple reasons: (1) Cells such as Tregs continually recirculate among blood, lymph, and tissue, such that blood sampling captures only partial information; (2) The graft microenvironment alters immune cell phenotype and function, providing contextual information absent from blood assays; (3) Traditional single-marker approaches are limited in capturing the complex dynamics of the immune network; (4) Interactions among immune components are difficult to discern in blood assays. For transplant follow-up, peripheral blood immune monitoring should be integrated with histologic examination, imaging, and clinical indicators, incorporating novel composite markers when necessary to enhance predictive accuracy. This also underscores that development of new immune monitoring tools should focus on integrating multiple information sources to bridge the disconnect between blood and tissue data (Figure 2).

## 8 Future perspectives

Having reviewed Treg dynamics across different post-heart transplant stages within a staged framework, along with their associations with rejection and long-term lesions, the next critical question is how this evidence can be translated into actionable follow-up assessments and intervention designs. The clinical significance of Treg-related readouts primarily manifests in three aspects: first, supplementing the evaluation of immunoregulatory status for risk stratification and mechanistic insights; second, explaining common discrepancies between peripheral blood and graft readouts to reduce misjudgments based on single markers; third, providing a foundation for the design of targeted immunoregulatory strategies. Accordingly, the following sections will sequentially discuss principles for combining and interpreting monitoring markers, as well as key variables and endpoint settings in Treg cell therapy.

### 8.1 Multimarker biomarker detection strategy

To translate Treg-related signals into actionable follow-up tools, this section proposes a comprehensive detection strategy based on four key dimensions: Treg quantity, function, effector/inflammatory responses, and tissue/endothelial injury. This approach integrates peripheral blood and tissue information within the same time window, thereby enhancing the discriminatory accuracy for rejection risk and immune tolerance status. Specifically, joint modeling of Treg quantity and phenotype, Treg function, effector T cell activation and expansion signals, and markers of endothelial activation and tissue injury can be employed, followed by stratified follow-up (110). Longitudinal trend analysis of these markers enables clinicians to implement intervention adjustments earlier, before immune imbalance or risk escalation occurs; simultaneously, for low-risk patients with stable markers, monitoring data can confidently support reduced biopsy frequency or gradual tapering of immunosuppressant doses (111). Overall, this multiparameter combination and trend monitoring approach provides a critical pathway toward precision follow-up and individualized immunoregulation.

To facilitate clinical implementation, we propose a tiered assessment strategy. For routine monitoring, we recommend a Minimum Assessment Panel comprising CD3, CD4, CD25, surface CD127, and intracellular *FOXP3*, which serves to provide a baseline estimate of Treg frequency and phenotype. In complex cases characterized by unexplained rejection or blood-graft discordance, an Advanced Assessment Panel is warranted. This comprehensive profile should include TSDR demethylation analysis and Helios expression to verify lineage stability, CTLA-4 and IL-10 to evaluate functional potential, and Th17-associated markers such as ROR*γ*t to detect cellular plasticity.

### 8.2 Treg cell therapy

Adoptive transfer of functionally intact Tregs to patients represents another frontier strategy for achieving immune tolerance. Given the scarcity of Tregs in peripheral blood and their competition with host effector T cells for limited survival resources, *ex vivo* expansion followed by adoptive transfer can rapidly provide recipients with large numbers of highly suppressive Tregs, thereby potentially rebalancing immune responses and promoting tolerance establishment (112). In animal models, infusion of donor-specific Tregs has repeatedly been shown to prolong graft survival and even induce long-term tolerance. In recent years, multiple phase I clinical trials in kidney transplantation have investigated the safety of autologous Treg therapy (113). Overall, infusion of autologous peripheral blood-expanded Tregs has been generally well tolerated in participants, with rare serious adverse events, although clinical benefits require further validation in larger, controlled studies. However, Tregs obtained from peripheral blood are limited in quantity, and expanded adult peripheral Tregs exhibit reduced phenotypic stability. To address this issue, recent innovations have emerged: one involves obtaining Tregs from discarded thymic tissue (thyTregs). In a method developed by Bernaldo de Quirós et al., large numbers of naïve Tregs are isolated from thymic tissue excised during pediatric cardiac surgery, expanded in short-term culture, and reinfused into patients (37). Thymus-derived Tregs are more naïve with high *FOXP3* gene demethylation, resulting in greater phenotypic stability and stronger suppressive function post-expansion. The second is the preparation of donor-specific Tregs. To enhance efficacy, some trials incorporate donor antigen stimulation during expansion, such as mixed lymphocyte reactions or stimulation with cells expressing donor HLA. The resulting Tregs are more donor-specific, theoretically providing more effective rejection suppression (114).

Current studies such as The ONE Study are testing Tregs from different sources and culture methods in kidney transplantation; although sample sizes are small, initial results indicate safety (115). Clinical experience with Treg therapy in heart transplantation remains very limited. On one hand, heart transplant patients are fewer than kidney recipients, increasing trial organization difficulty; on the other hand, frequent cardiac biopsies are not feasible due to arrhythmia risks, necessitating reliance on indirect endpoints for efficacy assessment (116). Nevertheless, the prospects for Treg therapy in heart transplantation remain promising.

### 8.3 Other intervention approaches

**Figure 3:**
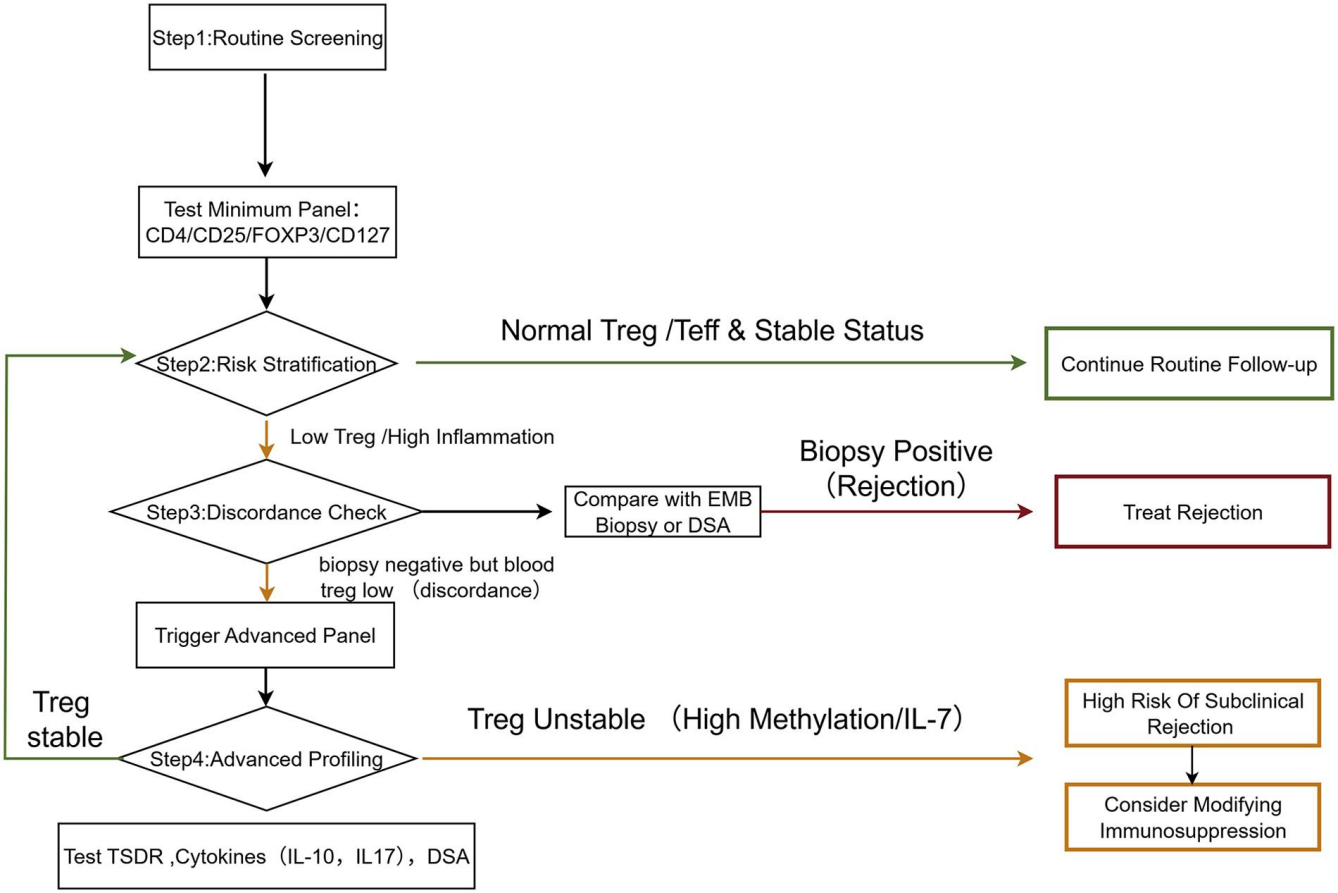
A proposed clinical workflow for Treg-assisted immune monitoring. This algorithm integrates peripheral blood Treg screening with standard endomyocardial biopsy (EMB) to guide clinical decision-making. Color coding indicates clinical status and workflow logic: Blue represents routine screening initiation; Green denotes a stable, low-risk status warranting standard follow-up; Orange highlights discordant results or potential subclinical risks necessitating advanced functional profiling; Red indicates confirmed rejection requiring therapeutic intervention; and Black arrows/lines represent the standard procedural flow between decision points.

A proposed clinical workflow for Treg-assisted immune monitoring after heart transplantation is shown in Figure 3. Intervention strategies can be categorized by their clinical readiness. First, optimizing current immunosuppressive regimens represents an immediately applicable approach. Second, adoptive Treg therapy is currently undergoing clinical trial evaluation for safety and efficacy. Finally, emerging experimental strategies, such as histone deacetylase (HDAC) inhibitors and gut microbiota modulation, offer potential future avenues for enhancing Treg stability.

Beyond the primary strategies discussed above, other related immunoregulatory pathways also warrant attention, such as modulation of cytokine pathways other than IL-2 to influence immune responses. Interventions targeting these cytokine signaling pathways offer potential therapeutic options for enhancing immune tolerance and suppressing rejection. IL-2 is the key cytokine for maintaining Treg survival and function, whereas cytokines such as IL-6, IL-7, and IL-15 predominantly promote expansion and activation of effector T cells (Teffs) and memory T cells. The IL-6 signaling pathway not only facilitates proinflammatory Th17 differentiation but also, through aberrant IL-6/STAT3 activation, suppresses Treg development and function, disrupting the Teff-Treg balance; the IL-7/IL-7R axis plays a crucial role in memory T cell survival and expansion, and blocking this signaling in experimental models can suppress antigen-specific memory responses. Based on this understanding of cytokine-dependent competition, blocking pathways that promote effector/memory T cells—such as IL-7 or IL-15—may indirectly enhance Treg dominance in immunoregulation by reducing competition for limited resources (117). Small-molecule drugs such as HDAC inhibitors can enhance *FOXP3* expression and Treg suppressive function by altering epigenetic states, and are thus considered promising immunoregulatory agents for inducing transplant tolerance. HDAC inhibitors promote *FOXP3* protein acetylation, thereby increasing its stability and target gene binding capacity, while also boosting Treg numbers and function; they have demonstrated enhanced immune tolerance in animal models of autoimmunity and organ transplantation (81).

Mounting evidence indicates that the host gut microbiome and its metabolites play a crucial role in modulating systemic immunity, with microbe-derived short-chain fatty acids (SCFAs) promoting the differentiation and function of Tregs. SCFAs, such as butyrate and propionate, act through multiple mechanisms—including epigenetic regulation and signaling pathways—to enhance the development and proliferation of *FOXP3*+ Tregs while influencing proinflammatory T cell subsets, thereby contributing to the maintenance of immune tolerance. Based on this fundamental immunoregulatory effect, gut microbiota modulation has been proposed as a potential approach to elevate Treg levels and immunoregulatory capacity in transplant recipients, warranting in-depth investigation in the context of transplant immunology (118).

When considering these immunoregulatory strategies, several key issues warrant particular attention. First is safety: any strategy enhancing immunoregulation may carry risks of broad immunosuppression, making it a major challenge to ensure that such regulation targets graft-specific immune responses without compromising anti-infectious or antitumor immunity. Notably, Treg-targeted therapies possess inherent specificity, as Tregs primarily maintain immune homeostasis by suppressing effector T cell responses, with relatively minimal direct impact on other arms of the immune system. Second, inter-individual variability represents another critical concern. Differences in recipient immune backgrounds may result in heterogeneous responses to Treg therapy. Patients with a history of autoimmunity may exhibit distinct Treg characteristics compared to the general population, necessitating personalized treatment regimens.

Finally, establishing robust evaluation systems is essential. These emerging therapies rely on appropriate biomarkers for efficacy assessment, particularly to determine whether infused Tregs successfully home to and exert suppressive effects within the graft. Despite persistent challenges, Treg-centered immunoregulatory strategies are driving transplant immune management toward more targeted and precise regulation, away from non-specific immunosuppression. As understanding of Treg biology deepens and advances in cellular and molecular engineering continue, future integration of immune monitoring and intervention strategies holds promise for precisely modulating post-transplant immune responses, thereby reducing rejection risk, minimizing complications such as infections, malignancies, and drug-related toxicities, and substantially improving long-term graft survival.

## 9 Conclusion

Tregs play a pivotal role in immune responses following heart transplantation, spanning the entire post-transplant immunological process. Their role is particularly crucial in restraining rejection, promoting long-term immune tolerance, and facilitating immunoregulation. Stage-specific analysis reveals that the early phase (0–30 days) is dominated by injury-related sterile inflammation, where Treg recruitment and its influence on local inflammation resolution and tissue repair may substantially alter risks of early immune injury and rejection onset; the intermediate phase (1–6 months) features persistent donor antigen stimulation and ongoing immunosuppression adjustments, with Treg quantity, phenotype, and suppressive function directly involved in regulating acute cellular rejection; and the late phase (>6 months) involves progressive accumulation of chronic low-grade inflammation and vascular remodeling, where Tregs suppress ongoing immune attacks while potentially influencing fibrosis and vasculopathy progression via repair- and remodeling-associated pathways. Therefore, comprehensively evaluating the balance between immunosuppressive effects and risks associated with tissue remodeling emerges as a key focus for future research.

Future immune management should adopt a staged follow-up framework, integrating Treg phenotype and function, histologic and molecular markers, drug exposure, and clinical events to construct a dynamically updated risk stratification model. This model will facilitate rejection risk assessment and guide the timing and intensity of immunosuppressant tapering or Treg-targeted interventions. Existing quantitative strategies, such as gene expression profiling, have been validated in specific populations for reducing biopsy frequency without increasing serious adverse outcomes, while non-invasive markers like donor-derived cell-free DNA have demonstrated potential value in monitoring graft injury and rejection, poised to become key input variables in future risk assessment models.

While this stage-specific framework is derived primarily from heart transplantation data, the fundamental principles—such as the impact of ischemia-reperfusion injury on early Tregs and the effects of immunosuppressants—may share commonalities with other solid organ transplants. However, organ-specific features, such as the unique pathology of cardiac allograft vasculopathy, necessitate tailored monitoring strategies.
